# Epidemic Erysipelas

**Published:** 1846-03

**Authors:** G. N. Fitch


### MEDICAL INTELLIGENCE.

Epidemic Erysipelas.—In the course of Lectures on the Theory and Practice of Medicine in Rush Medical College, Prof. Fitch presented to the class the results of his observations of the above mentioned disease. His remarks were based upon the analysis of 213 cases of the various forms of Erysipelas, of which he had kept accurately recorded notes, and 20 cases of Puerperal Peritonitis coexistent with the epidemic. We have been favored with a communication embracing the important deductions from this list, but from its length must postpone it until the first number of our next *enlarged* volume. Meantime we give a few notes taken down from Dr. F.'s lectures.

The Prof. at first doubted the contagiousness of the epidemic, but subsequently had abundant reason to change his opinion, and besides subscribing to the fact of its propagation by contact, proximity and innoculation, advances the *opinion*, which he thinks will be substantiated by others, that one attack confers immunity against a second, as in the case of other contagious eruptive fevers. He also acknowledges the identity of the Epidemic Erysipelas, and the concomitant cases of Puerperal Peritonitis.

In Prof. F.'s hands, no one point in the treatment appeared as effectual as free bleeding in the onset of the disease. A short delay in the use of the lancet, or its omission, materially increased the chances of a fatal issue. The Prof.'s favorite local application to the external inflammation was the liniment of linseed oil and lime water, and was much preferred after the use successively of the innumerable topical applications recommended by the various writers upon the subject. After effusion had occurred beneath the integuments, free incisions, when the nature and situation of the parts would admit of it, or otherwise, punctures to give exit to the serum, were almost invariably successful, and the only means of preventing suppuration, sloughing, and their horrible train of consequences. In the simple anginose varieties mercurials were of no perceptible advantage. The application topically of diluted tinct. of iodine, and administration of salines, was in these instances the treatment most applicable.

The Prof. in his remarks upon the puerperal cases, advanced the idea that the seizure might be prevented by sligh ptyalism, induced by the use of blue mass, in small doses, for three or four weeks before anticipated delivery. Several cases were cited in which it could not be doubted that this treatment had proved successful. Should this be substantiated by future observers, its value would be incalculable.

